# Supplementary material for: Effects of breastfeeding on postpartum weight change in Japanese women: The Japan Environment and Children’s Study (JECS)
Source: PLoS One. 2022 May 4;17(5):e0268046. doi: 10.1371/journal.pone.0268046 (PMC9067657; doi:10.1371/journal.pone.0268046)
Supplement: Attachment — Submitted filename: Response to Reviewers.docx [file pone.0268046.s001.docx]

February 28, 2022

Dr. Linglin Xie

Academic Editor

*PLOS ONE*

Dear Dr. Linglin Xie,

Thank you very much for your review of the manuscript (PONE-D-21-38089) that we sent on December 1, 2021. We wish to express our appreciation to the reviewers for their insightful comments on our paper. We have addressed each of the points you raised and revised the manuscript accordingly.

Responses to the Comments by the reviewer #1:

*1. The article suggested in the discussion that the combined effect of breastfeeding and controlling weight gain during pregnancy could be more helpful for weight loss in overweight and obese population. However, Table 2 and 3 barely showed a significant effect of FB on weight loss and weight retention. Why will a combined method work?*

Reply: We apologize for our insufficient explanation of combined effect. As shown in table 3, there was less weight retention in FB than in NFB in overweight and obese groups (overweight: FB −2.2 kg, NFB −0.7 kg, obese: FB −4.8 kg, NFB −3.4 kg). In addition, as shown in table2, FB had more weight loss than NFB (overweight: FB 10.6 kg, NFB 9.1 kg, obese: FB 10.0 kg, NFB 9.1 kg). It was reported in previous study that less weight gain during pregnancy leads to less weight retention. In present study, as shown in table1, overweight and obese had less weight gain during pregnancy than the other groups. Therefore, we discussed that breastfeeding and the restriction of weight gain during pregnancy resulted in more weight loss and less weight retention in overweight and obese women, which we described as a combined effect in the discussion (lines 238-249).

*2. In the discussion, the author indicates that the weight retention in the underweight group with FB is also lower than NFB; while the weight loss increased in the FB group. However, this means that with BF, underweight mothers lose more weight than NFB. Is this concerning? Will it be more beneficial for underweight mothers to gain weight postpartum?*

Reply: Thank you for your comment. In general, there is an increased risk of low birth weight and preterm delivery in prepregnant underweight women. We added this consideration because the risk of the following pregnancy will be more increased if prepregnant underweight women lose even more weight due to breastfeeding. However, on average, underweight FB women did not lose more weight than their pre-pregnancy weight, therefore we concluded that underweight women can breastfeed with　no problems.

*3. Lastly, I’m wondering what if the author separates the NFB group into the mixed group and the artificial feeding group? Will the mixed feeding method show any beneficial outcome compared to both groups?*

Reply: Thank you for the suggestion. As suggested, we believe the results when NFB is divided into full formula milk and mixed feeding would be valuable. However, the participants who fed only formula was very few in JECS study, therefore we considered it inappropriate to analyze complete formulas and mixed feeding separately. We hope you understand this situation.

Responses to the Comments by the reviewer #2:

*1. In the methods section (line 97), the exclusion criteria for weight include those outside of the range 25-300kg. Is this the true range, or a typo? It seems that 300kg is extremely heavy.*

Reply: We appreciate helpful comments of Reviewer #2. The reviewer's comment is correct. We reviewed the subjects again and found that there were no participants over 200 kg in the analysis, therefore we revised the upper limit of the range to 200 kg (line 99).

*2. Do you have any information about if the weight loss from the FB overweight and obese mothers lost enough weight to improve their pre-pregnancy BMIs? For example, was an FB obese mother's postpartum weight loss enough to improve their BMI to an overweight status?*

Reply: We think it is natural for the reviewer to question this point. Although this study was able to show that breastfeeding resulted in greater maternal postpartum weight loss, the effect of weight loss was not sufficient to improve the BMI category. It would be more effective to use diet or exercise therapy in addition to breastfeeding.

*3. Do you know if the women were able to retain their weight loss after they were finished breastfeeding? I'm wondering if the body condition of these mothers that lost a great deal of weight postpartum was retained even 1-2 years after delivery.*

Reply: We agree that additional information on duration of breastfeeding effect as the reviewer suggested would be valuable. Regrettably, however, because the observation period of maternal weight is up to 6 months after delivery in JECS study, we are unable to do the additional analysis. We have included your point as a consideration for future study.

*4. There is several instances of switching tenses (i.e. from third to first person point of view) in the writing style. Converting all of these sentences to read in third person would help the manuscript read better.*

Reply: We apologize for the difficulty in reading the manuscript. Since our native language is not English, we have asked the experienced English-native editor to edit this manuscript again.

We believe that we have addressed reviewers’ comments and hope that the revised manuscript is now acceptable publication in *PLOS ONE*. Thank you for your generous consideration.

Sincerely,

Shigeru Aoki

Perinatal Center for Maternity and Neonate, Yokohama City University Medical Center Yokohama, Kanagawa, Japan.

4-57 Urafunechou, Minami-ku, Yokohama, Kanagawa, 232-0024, Japan

email: smyyaoki@yahoo.co.jp
